# A predictive model of inappropriate use of medical tests and medications in Bronchiolitis

**DOI:** 10.11604/pamj.2020.37.94.22712

**Published:** 2020-09-25

**Authors:** Jefferson Antonio Buendía, Carlos Andrés Rodríguez

**Affiliations:** 1 Grupo de Investigación en Farmacología y Toxicología (INFARTO), Universidad de Antioquia, Medellín, Colombia,; 2 Department of Pharmacology and Toxicology, School of Medicine, University of Antioquia, Medellin, Colombia,; 3 CIEMTO *[drug and poison research and information center]* at Integrated Laboratory of Specialized Medicine (LIME), Facultad de Medicina-IPS Universitaria, Universidad de Antioquia, Calle 64 #51-31, 050010, Medellin, Colombia

**Keywords:** Bronchiolitis, cost, resources, Colombia

## Abstract

Few studies have identified predictors of inappropriate use of medications and medical tests in bronchiolitis. This study aimed to look for potential factors associated with the inappropriate use of medications and tests in bronchiolitis. A retrospective study that included all infants under two years of age in tertiary center admitted due to Bronchiolitis from January 2015 to December 2018. We defined a composite score as the main outcome variable. 1930 patients were included. The most prescribed medications were nebulized hypertonic saline in 1789 patients (92.6%), albuterol (56%), and β-lactam antibiotics (26.4%). The medical tests more commonly ordered were hemogram (95.9%), chest X-rays (92.2%) and C-reactive protein (79.8%). After controlling for potential confounders, it was found that the length of hospital stay increases the risk of the inappropriate use of medications and tests (OR 1.29; CI 95% 1.01-1.65), whereas fever (OR 0.22; CI 95% 0.06-0.71) and leukocytosis (> 15,000/μL) (OR 0.09; CI 95% 0.03-0.32) at admission decrease the risk of the inappropriate use of medications and tests. Inappropriate use of diagnostic tests and drugs for bronchiolitis was a highly prevalent outcome in our population. Patients with longer hospitalizations, absence of fever and a normal white blood cell count at admission, were at increased risk of inappropriate use of medications and medical tests.

## Introduction

Bronchiolitis is the most common respiratory infection in children < 1 year of age and is the main cause of hospitalization in the youngest children, especially during the winter season. Although several clinical practice guidelines have been developed [1, 2], there is significant use and overuse of medications and medical tests with insufficient evidence of effectiveness, generating unnecessary and costly expense of resources without improvement in clinical outcomes [3-5]. This phenomenon is accentuated in patients with bronchiolitis, previous reports indicate that more medical tests are ordered to these patients and they receive more drug prescriptions [6, 7]. Recent studies have identified atopic dermatitis, a longer hospital stay, and number of siblings as independent predictors of inappropriate management of bronchiolitis [8, 9]. However, these studies are limited because they did not include patients treated in the emergency department, the setting where most episodes of bronchiolitis are treated; limiting their external validity. It is important to adequately identify predictors of inappropriate use of medications in bronchiolitis to plan risk-based strategies and thus optimize the use of health resources. Implementing strategies to reduce the inappropriate use of medications that lack robust evidence leads to effective use of health care services and avoids resource wasting, without reducing service quality and improving disease outcomes. This study aimed to identify potential factors associated with the inappropriate use of medications and tests (IUMT) in bronchiolitis in a population of infants hospitalized in a tertiary level center in Colombia.

## Methods

A retrospective study that included all infants under two years of age in tertiary centers, in Rionegro, Colombia due to bronchiolitis (ICD-10 code: J21.0, according to the national clinical guideline of bronchiolitis [1]) from January 2015 to December 2018. Rionegro is a city in Antioquia, Colombia, located in the sub region of Eastern Antioquia, at an average elevation of 2,125 meters above sea level. The municipality of Rionegro had a total population of 101,046 inhabitants, the sixth largest populated area in Antioquia, with two tertiary referral hospital [10]. The study protocol was reviewed and approved by the Institutional Review Board of Clinica Somer (No 281015) and the University of Antioquia (No 18/2015). After reviewing the electronic medical records, we collected the following variables: age, sex, weight, height, signs, and symptoms at admission (e.g. fever, chest in drawing, chest auscultation abnormalities like rhonchi or crepitation), history of prematurity, broncho pulmonary dysplasia confirmed by a specialist physician at the discharge from neonatology unit, comorbidities (congenital heart disease, neurological disease), results of chest X-rays or other medical test, drugs and other treatments, and complications (pneumonia, atelectasis, sepsis, ICU).

A composite outcome score was used to define inappropriate use of medication. This composite outcome score aggregated the use of diagnostic tests (hemogram, C-reactive protein, urine culture, chest radiography, and blood cultures or other tests) and the use of medications: use of bronchodilators (inhaled or nebulized β_2_agonists, inhaled or nebulized anticholinergic, and nebulized epinephrine), corticosteroid therapy (inhaled or systemic) and antibiotics. The use of nebulized hypertonic saline was excluded because national guidelines for bronchiolitis allow its use in order to reduce the length of hospital stay [1]. Each of these items had a binary score: 0 indicating that the medication or test was not used and 1 indicating that they were used. The use of corticosteroids, inhaled β_2_agonists and epinephrine was deemed as appropriate only if they were continued after a positive clinical response to a monitored trial. The use of antibiotics was deemed as appropriate in patients with confirmed bacterial infections (blood culture, urine culture or chest X-rays with lobar consolidation). The total score was calculated by adding up the scores, ranging from 0 to 8 (best to worst). A composite outcome score of β_2_was designated as “inappropriate use of medications and medical tests”.

**Statistical analysis:** continuous variables are presented as mean ± standard deviation (SD) or median ± interquartile range (IQR), whichever is appropriate. Categorical variables are showed as numbers (percentage). Differences between continuous variables were analyzed using the unpaired t-test or Wilcoxon's signed-rank test, whichever was appropriate. Associations between categorical variables and the outcome were analyzed using the β_2_test or Fisher's exact test, whichever was appropriate. We used logistic regression models to adjust for potential confounding variables. All statistical tests were two-tailed, and the significance level used was p < 0.05. The data were analyzed with the statistical package Stata 15.0 (Stata Corporation, College Station, TX). The study protocol was reviewed and approved by the ethics boards of Clinica Somer (No 281015) and the University of Antioquia (No 18/2015).

## Results

During the study period, 1930 patients had a diagnosis of bronchiolitis. The mean age was 5.2 months (range 0.09 to 24 months), 19% patients were younger than 1 month and 69% were younger than 6 months of age. 58.5% were males. Fourteen percent had a history of prematurity and 7% a significant cardiac or chronic neurological comorbidity. The average hospital stay lasted for 5.2 days. Severe hypoxemia (SatO2 ≤ 90% in the emergency department) was resent in 59.5% of the patients. 12.9% patients had pneumonia, and.7% patients experienced sepsis. The medications most often prescribed were nebulized hypertonic saline in 1789 patients (93.3%), albuterol (56%), epinephrine (34.7%), β-lactam antibiotics (26.4%) and systemic corticosteroids (11.9%). Ninety-six percent of β-lactam antibiotics, 90% of bronchodilators, 86% of corticosteroids and epinephrine, were classified as inappropriate. The medical tests more commonly ordered were hemogram (95.9%), chest X-rays (92.2%) and C-reactive protein (79.8%). Seventy-four percent of hemograms, 72% of chest X-rays and 40.4% of C-reactive protein, were classified as inappropriate. Multivariate analyses were conducted to determine independent predictors of IUMT. After controlling for potential confounders, it was found that the length of hospital stay increases the risk of IUMT [OR 1.29; CI 95% 1.01-1.65], while fever [OR 0.22; CI 95% 0.06-0.71] and leukocytosis [> 15,000/μL] [OR 0.09; CI 95% 0.03-0.32] at admission decrease the risk of UIMT (Table 1).

**Table 1:** predictors of inappropriate use of medications and medical tests in RSV-related bronchiolitis through multivariate analysis

Variable n (%)	OR	95% CI	p value
Length of hospital stay	1.29	(1.01-1.65)	**0.038**
Fever	0.22	(0.06-0.71)	**0.012**
Comorbidities (CHD neurological)	3.35	(1.25-43.59)	0.355
Leukocytosis (> 15,000/mL)	0.09	(0.03-0.32)	**0.000**
Age (months)	1.00	(0.91-1.10)	0.910

CHD: congenital heart disease

## Discussion

The results of this study show that there was a high prevalence of inappropriate use of medications and medical tests in bronchiolitis. Participants with longer hospital stay were at increased risk for inappropriate use of drugs and diagnostic tests, while patients without leukocytosis and fever had reduced risk for inappropriate use. We identified a set of predictors independently associated with inappropriate use of medications in bronchiolitis not previously reported in the literature. Greenough *et al*. found that infants with bronchiolitis with RSV infection required more prescriptions (p=0.004) and more respiratory medications (p=0.0044) with a higher cost of care than a patient without bronchiolitis (p=0.0068), but did not identify the variables associated with this higher use of medications and tests in bronchiolitis [6]. In bronchiolitis, independent of the etiologic agent, some features related to the inappropriate use of medications and diagnostic tests have been determined. Sarmiento *et al*. found that infants with bronchiolitis and a personal history or diagnosis of atopic dermatitis [OR 5.30; CI 95% 1.14-24.79; p = 0.034], more extended hospital stay [OR 1.48; CI 95% 1.08-2.03; p=0.015], and a higher number of siblings [OR 1.92; CI 95% 1.13-3.26; p=0.015], were at increased risk for inappropriate use of diagnostic tests and treatments [5]. However, the study did not include patients treated in the emergency department, which limits its external validity.

In our study, we identified that, as expected, patients with longer hospitalizations have a higher risk to undergo unnecessary tests or receive unneeded medications, a finding in agreement with previously published papers in bronchiolitis [5]. These findings highlight the need to implement early discharge strategies or home hospitalization as ways to rationalize medical spending, as has been proved in other diseases [11]. It is very striking how specific findings, such as the absence of fever, place the doctor in doubt about the diagnosis of bronchiolitis. The irrational prescription of antibiotics is nothing new in bronchiolitis. In the late 1990s this practice was well documented [12], and despite the implementation of clinical practice guidelines it still continues in most countries [13]. On the other hand, current evidence-based guidelines are clear in not recommending chest X-rays to all patients with bronchiolitis, not only because they do not change medical behaviors but also because they are not cost-effective [7].

Chest X-rays are only indicated in patients where complications secondary to bronchiolitis are suspected such as atelectasis, bacterial pneumonia or pneumothorax [1, 2]. Our study has the following limitations: since it was based on medical records review, assessing the appropriateness of the interventions was based on the information registered in them. Some patients may have had additional findings that would have altered the appropriateness of disease management. However, all associated variables in previous models were included to minimize the information bias. Second, our study was limited to patients hospitalized at a single tertiary care pediatric center, and our findings could not be generalizable to all patient with bronchiolitis. However, we hope that our results represent an initial step toward designing interventions that include a more rational approach to the management of patients with bronchiolitis.

## Conclusion

Inappropriate use of diagnostic tests and drugs for bronchiolitis was a highly prevalent outcome in our population. Patients with longer hospitalizations, absence of fever and a normal white blood cell count at admission, were at increased risk of inappropriate use of medications and medical tests.
